# Spatial perspective-taking abilities on ambiguous tactile stimuli: The case of children with autism spectrum disorder or developmental coordination disorder

**DOI:** 10.1371/journal.pone.0355395

**Published:** 2026-09-21

**Authors:** Jean Xavier, Armand Verley, David Cohen, Hugues Pellerin, Louise P. Kirsch, Malika Auvray

**Affiliations:** 1 Department of Child and Adolescent Psychiatry, Henri Laborit Hospital Center, Poitiers, France; 2 CNRS UMR 7295, Cognition and Learning Research Center, University of Poitiers, Poitiers, France; 3 Department of Child and Adolescent Psychiatry, Pitié-Salpêtrière Hospital, Paris, France; 4 Institut des Systèmes Intelligents et de Robotique, ISIR, Sorbonne Université, CNRS, Paris, France; 5 Integrative Neuroscience and Cognition Center, INCC, UMR 8002, Université Paris Cité, CNRS, Paris, France; BSMHFT National Centre for Mental Health, UNITED KINGDOM OF GREAT BRITAIN AND NORTHERN IRELAND

## Abstract

Spatial perspective-taking corresponds to the ability to adopt someone else’s point of view. Abnormalities in visual perspective-taking have been reported in neurodevelopmental disorders (NDDs), such as Autism spectrum disorder (ASD) and developmental coordination disorder (DCD), which are highly comorbid conditions. This study explored the developmental aspects of perspective-taking in the tactile modality in typically developing children and children with NDD. A total of 107 children, aged between 6 and 17 years, completed the study: 74 were typically developing (TD) and 33 had a neurodevelopmental disorder (NDD) (24 had ASD and 9 had DCD). We used a graphesthesia task to investigate the perspectives adopted by children when interpreting ambiguous tactile symbols (b, d, p and q) presented on their stomach. The children’s responses to the stimuli allowed us to infer the perspective they had adopted to interpret them: egocentric (head- or trunk-centred) or decentred. The spontaneous perspective was predominantly egocentric (82% and 77% for TD and NDD groups, respectively). There were no significant differences between the groups. During S2 and S3, in which we imposed a perspective on participants, accuracy increased with age for both groups. Additionally, the NDD group demonstrated significantly lower accuracy than the TD group in session 2. Response times (RT) were similar, except for the imposed decentered perspective. RT was significantly higher in the NDD group than for the TD group. From a developmental perspective, our findings highlight the embodied strategy involved in perspective-taking in the tactile modality, as well as its specificities in children with NDD. We discuss our results in relation to possible impairments in developmental dimensions, including inhibitory control and multisensory integration amongst those with ASD.

## Introduction

According to the embodied cognition framework, many aspects of intersubjectivity are rooted in the interactive experience between the body and the perceivable environment [1]. These experiences involve various mental spatial transformations that occur within two main perspectives: egocentric (e.g., the perception from our body) or decentred (relative to the ability to adopt another spatial perspective) [2,3]. The decentred perspective is associated with a person’s ability to consider someone else’s point of view [4–9]. When this point of view relates to spatial coordinates, it is called perspective-taking (PT).

Research on visuospatial skills suggested two levels of visuospatial PT [9,10]. Level-1 PT abilities emerge at 2 years of age [11]. It corresponds to the ability to judge what a person can and cannot see without changing the vantage point, in other words, whether an item is occluded or not from their line of sight. Level-2 PT starts to appear around 4–5 years of age [12]. It corresponds to the ability to understand that two different people viewing a scene or object simultaneously do not necessarily see objects in the same way [10]. Level-2 PT corresponds to the ability to mentally imagine a scene from an external viewpoint.

Mental rotation and embodied perspective taking are two distinct cognitive strategies for Level 2 VSPT [13]. The latter requires the participant to undergo an embodied spatial transformation, shifting it from their own to that of the target [11,13] as in the classic Three Mountains Task [14]. Piaget & Inhelder (1967) [14] used a construction model of three mountains and a doll. Children between the ages of 4 and 11 were asked to answer whether the doll could see or not particular objects, which were sometimes hidden behind the layers of mountains. The results indicated that before the age of 9–10, it was difficult for children to fully overcome their egocentric visuospatial biases and make judgments from another person’s perspective [15]. This result supports the view that, while adopting an egocentric perspective is immediate and automatic, taking a decentered perspective demands cognitive effort. The spontaneous shift from an egocentric to a decentered perspective is rooted, in part, in the growing efficiency to inhibit the first-person perspective [16]. It progressively develops from age 10 with the acquisition of mapping and spatial navigation abilities [17–19].

Abnormalities in PT have been described in neurodevelopmental disorders (NDD) such as Autism spectrum disorder (ASD) and developmental coordination disorder (DCD) [20–22]. ASD are characterized by deficits in social communication, social interaction, and stereotyped patterns of behavior, interests, or activities [23]. This condition includes a wide heterogeneity in either descriptive or etiological levels (for a review, see [24,25]). Children with DCD display motor incoordination and visual-spatial impairments [22].

Most studies exploring visuospatial PT suggest that whilst level-1 PT is intact in people with ASD, level-2 may be impaired [26]. This can be attributable to the fact that the ability to perform own-body transformation (i.e., egocentric transformation) is deficient in people with ASD but not their ability to perform mental rotations (object-based transformations) [20,27]. A key ASD cognitive feature is atypical perceptual processing [20,28–31]. This atypicality involves sensory perception and multisensory integration impairments [32,33]. Concerning tactile perception in children with autism, studies have yielded conflicting results: some studies have found alterations in touch perception, which were not confirmed in other studies (for a review see [34]). These results can be explained by the clinical heterogeneity in autism and by the diversity of tasks that were used. Using vibro-tactile tasks, Leekam et al. (2007) [35] and Tomchek and Dunn (2007) [36] found altered tactile perception in early childhood with ASD in comparison with typically developing children. In addition, children with DCD often display visuo-spatial processing deficits [37], which underlie the difficulties in performing imagined transformations from an egocentric perspective [38].

Due to their high rates of comorbidity and frequent overlapping symptoms, it is difficult to consider ASD and DCD as distinct diagnostic categories with respect to spatial cognition and PT [39,40]. PT is a complex embodied process that involves the integration of a multimodal sensory input [3,41–43]. However, the majority of studies, either for typically developing children or for children with NDD, focused on the visual component of PT [20]. Fewer explored PT when the input modality is in another sensory modalities, such as touch, which brings specificities. In particular, in touch, a perceived stimulation can be interpreted either from i) an egocentric perspective, which is centred either on the stimulated surface or on the head, or 2) a decentered perspective [44].

**Fig 1:**
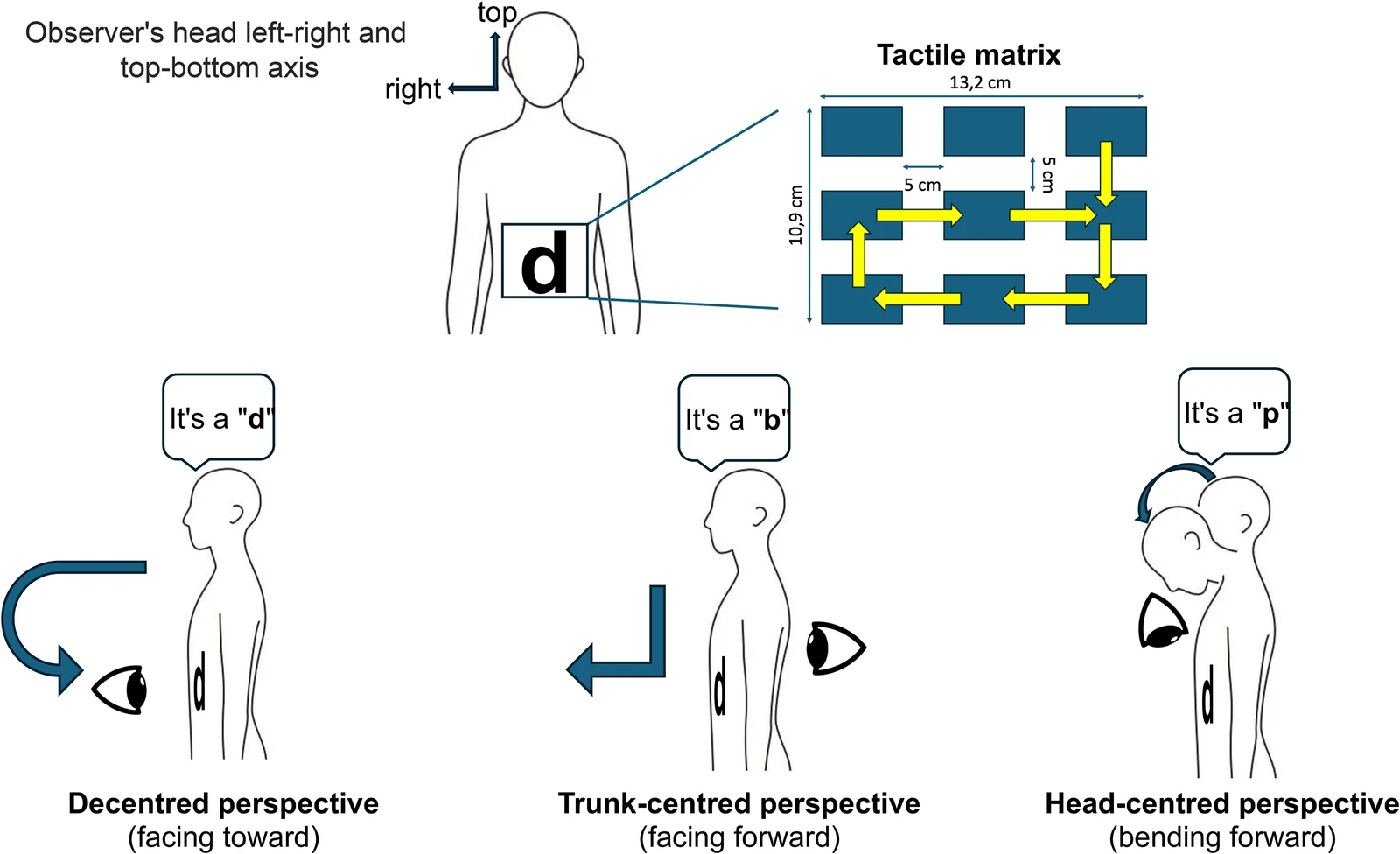
Schematic illustration of the 9 (3*3) array of rectangular vibrators. The sequence of vibrations that is represented corresponds to the drawing of the letter d. Illustration of the three possible perspectives that can be adopted on this tactile letter d.

To the best of our knowledge, the developmental aspects of PT in the tactile sensory modality have not yet been addressed in typically developing (TD)children or children with NDD. Our exploratory study focused on the developmental aspects of PT in children with NDD (both ASD and DCD), as compared to those of a group of TD children. We used the graphesthesia task developed by Arnold et al. (2016, 2017) [41,44], which provides a tool to investigate the embodied nature of spatial PT in the tactile domain. In this task, participants had to interpret tactile symbols (e.g., the ambiguous letters b, d, p, and q) presented on their stomach. The same perceived stimulation can be interpreted as corresponding to different symbols as a function of the perspective that is taken when interpreting the stimulation (see Fig 1). In session 1, the participants were asked to report the letter as it feels the most natural to them, which allows us to infer their spontaneous perspective. In session 2, they had to change their spontaneous perspective to adopt an imposed one (which is opposed to their spontaneous one), involving a cost of changing perspective. In session 3, the participants were asked to return to their spontaneous perspective.

We made the following hypotheses: (1) As a spontaneous perspective, children would preferentially adopt an egocentric perspective (either a trunk-centred or a head-centred perspective). (2) When they are required to adopt an imposed perspective in session 2, their performance will decrease compared to session 1, signaling a cost. In addition, their recognition performance would increase with age as their inhibition skills do. (3) When returning to their spontaneous perspective (in session 3), their recognition performance will be higher than in session 2, and additionally will correlate with age. (4) Overall, children with NDD will have lower performance then TD children.

## Materials and methods

### Participants

107 children, aged between 6 and 17 years, took part in the study, involving 74 children with TD and 33 children with NDD. In the NDD group, 24 children were diagnosed with ASD and 9 children with DCD (see Table 1). Children completed the experiment from 18/12/2019–05/07/2024.

**Table 1:** Description of the participants.

Characteristic	TD N = 74	DCD N = 9	ASD^2^ N = 24
Age (years)	10.74 (1.99)^1^ [7.00–14.00]	11.56 (2.83)^1^ [8.00–17.00]	11.74 (2.88)^1^ [6.00–16.00]
Unknown	0	0	1
Sex			
Female	42 (57%)	1 (11%)	4 (17%)
Male	32 (43%)	8 (89%)	20 (83%)

^1^Mean (SD) [Min-Max]; n (%).

^2^One participant had both diagnosis: ASD and DCD.

The inclusion criteria for patients with NDD were a diagnosis of DCD or a diagnosis of ASD [45]. Exclusion criteria were ongoing medical conditions (e.g., seizures, sensory deficit) and severe language impairment that can be comorbid with both ASD and DCD. For each patient, diagnoses were based on all available information (interviews, family history data, and medical records) and made according to DSM-5 TR criteria [23]. The various assessments for ASD and DCD diagnoses are part of the standard care of our patients within the framework of their treatment. In this context, each patient was involved in a series of clinical evaluations. For ASD children: a questionnaire for evaluating autistic symptoms, the ADI-R [46]; a calculation of intellectual quotient using the WISC-IV (Wechsler Intelligence Scale for Children-IV) [47] or the WISC-V (Wechsler Intelligence Scale for Children-V) [48]. Children with DCD were evaluated during a psychomotor assessment that included quantitative testing (e.g., the Movement Assessment Battery for Children, M-ABC) [49] performed by an occupational therapist and they also completed theWISC-V (Wechsler Intelligence Scale for Children-V).

Patients from the NDD group were recruited in Paris and Poitiers, respectively from: 1) the Department of Child and Adolescent Psychiatry of the Pitié-Salpêtrière University hospital and 2) the Department of Child and Adolescent Psychiatry of Laborit Hospital in Poitiers.

Participants in the TD group attended typical schooling. They were recruited from 2 schools and 1 middle school in Poitiers. All the parents were informed, by means of an information letter, about the procedure of the study and its objectives. They all completed a written consent form authorizing their child to take part in the study. The processing of the collected data was validated as compliant by the ethics committee (National number IRB: 00012018–26).

### Apparatus

Tactile stimuli were presented using 9 rectangular vibrators (Haptuator Mark II, Tactile Labs, Montreal, Canada), which were arranged in a 3 by 3 array with a center-to-center spacing of 5 cm (see Fig 1). A nine-channel amplifier controlled each vibrator independently at a frequency of 250 Hz. The vibrator array was placed on the participant’s abdomen symmetrically with respect to their body’s mid-sagittal line. A layer of thin clothing was allowed between the skin and the vibrators. Participants individually selected the intensity of each vibrator using an adjustment method, which seems all the more important for a population with tactile particularities as reported in people with ASD and DCD [50]. Under no circumstances could the vibration be painful (the vibration frequency is 250 Hz, the vibrators are driven like speakers at 3 V • 0.5 A • 5.5 Ω).

### Stimuli

The stimuli consisted of the lowercase letters b, d, p, and q (see Fig 1). Tracing the letters involved a sequence of 8 vibrotactile stimuli mapping the trajectory of vibrations as if the letters were traced starting from their stems. The same tracing order was used for each letter, instead of following normal handwriting conventions, because the order in which the various strokes are made in normal manual drawing could have cued which letter was traced [51]. Note that it could be argued that tracing letters from the tail end could bias participants’ responses toward the letter “b”, given that only it is the only letter written this way. However, in the original experiment using this paradigm, each letter was reported equally by participants (24.8% of trials for b, 24.9% for d, 25.5% for p, and 24.8% for q; F (3, 237) <1, ns) [42]. Furthermore, before the experimental session, the participants were explained that the letters will always start from their tail. Each vibration lasted 250 ms with no interval between consecutive vibrations. This resulted in each letter being presented for a total duration of 6 s.

### Procedure

The procedure was the same as that used in the studies conducted by Auvray’s group [7,41,44,52,53] except for minor amendments to adapt the task to our population of children: a lower number of trials and answers up to 6 s after the end of the stimulation. The participants were instructed to avoid looking at the stimulated surface and to maintain a standing position with their heads facing forward (see Fig 1). Participants were asked to report the letter they felt on their stomach. The experiment lasted approximately one hour and consisted of three sessions following a training period. In Session 1, the spatial perspective adopted by participants was free. In particular the participants were instructed to report the letter as it seems the most natural to them. In session 2 a different spatial perspective to that adopted in Session 1 was imposed. Specifically, when a child spontaneously adopted an egocentric perspective in S1, the experimenter used a wooden letter to demonstrate how the child had read the letter spontaneously and explain that they now have to read it differently, i.e., from a decentred perspective. The experimenter told the child: “*Imagine that the letter is drawn on your stomach, just like on a T-shirt, and you are looking at it as someone standing in front of you would see your T-shirt*”. The experimenter then stood in front of the child and placed the letter on their own stomach, asking, “*Can you see the letter b, d, p or q (depending on the example used) on my stomach?*” Then, the experimenter placed the letter on their own stomach and said to the child, “*If you were in my place, you would also see the letter b, d, p or q (depending on the previous example) on your stomach.*”

Conversely, when the child spontaneously adopted a decentred perspective in S1, the experimenter used the same wooden letter to demonstrate how they had read the letter spontaneously and explained that they now had to read it differently, from an egocentric perspective. The experimenter said “*You can imagine the letters from your own point of view, as if you were standing on your stomach looking at the letter in front of you*”. Then the experimenter placed the wooden letter on their own stomach in front of the child and said: “*You will then recognise the letter b, d, p or q (depending on the example)*”.

In session 3, the same spatial perspective as in Session 1 was imposed (see Table 2).

**Table 2:** The possible free and imposed spatial perspectives in each session.

SESSION 1 Free perspective	SESSION 2 Imposed perspective	SESSION 3 Imposed perspective (same as S1)
Trunk-centred	Decentred	Trunk-centred
Head-centred	Decentred	Head-centred
Decentred	Trunk-centred	Decentred

At the start of Session 1, participants completed a training period consisting of one presentation of each letter. They were free to adopt the perspective that felt the most spontaneous to them to recognize the letters. No feedback was given on their response. Then, the experimental session was made of 3 blocks of 12 trials (3 presentations of each of the 4 letters in each block). This session aimed at defining the participant’s spontaneous perspective. Spontaneous perspective is defined as the perspective the most frequently freely adopted by a participant. When no perspective exceeded 33% at the end of the first 3 series, a 4th series was proposed to the child, then the preferential perspective was recalculated on series 2, 3, and 4. If no perspective emerged, the child was given a letter recognition exercise to verify that the problem was not due to an insufficient knowledge of the letters. If these tests were successful, the child was asked to complete a 5th series, and the spontaneous perspective was computed on series 3, 4, and 5. If no perspective was superior to 33%, the experiment stopped. If a spontaneous preferential perspective emerged, the child proceeded to Session 2.

Then, the participants completed session 2, for which they received instructions to adopt a specific perspective different from their spontaneous one. Session 2 was preceded by training period during which the child received feedback on the correctness of their responses after each trial. A first block of 12 trials (3 presentation of each of the letters) was presented to the participants. Then, if this was not enough, the child could train this way as much as they wished but the participants overall completed 1 or 2 blocs. Then, the experimental session consisted of two blocks of 12 trials. No feedback was provided during the test blocks, but the participants were informed of their percentages of expected responses and mean response times at the end of each block.

In Session 3 (S3), the participants received instructions to return to their spontaneous perspective. This session was preceded by a training similar to that of session 2. In the experimental session, the participant were presented with two blocks of 12 trials.

During all sessions, the participants responded by pressing a key on the computer keyboard with their index finger of their dominant hand. The keys c, v, b, n were renamed with stickers b, d, p, q, respectively, to avoid searching for the letter on the keyboard. Participants were instructed to keep their head straight while the letters were traced, so that they could not see their abdomen. They were also told to respond as accurately and quickly as possible, and at any time from the beginning of the first vibration and up to 6,000 ms after the last vibration ended. Accuracy was emphasized over speed. After these 6,000 ms, there was a 4,000 ms interval before next trial began.

### Metrics

The following four metrics were defined for subsequent analyses:

*Spontaneous perspective* is defined as the perspective most frequently adopted freely by a participant in session 1 (trunk-centred, head-centred, or decentred). If two perspectives were equally frequent, no spontaneous perspective was assigned.

*Dominance of the spontaneous perspective* is defined as the difference in frequency between the spontaneous perspective and the second most frequently adopted perspective. This index ranges from 0 (excluded) to 1, with values near 0 indicating two perspectives in competition and values close to 1 indicating an exclusive preference.

*Accuracy* is defined here as the proportion of correctly adopted perspectives in sessions 2 and 3, i.e., the ability to identify the correct letter according to the expected perspective (see Table 2).

*Response time (RT)* is defined here as the time in milliseconds taken to adopt a perspective in sessions 2 and 3, regardless of it corresponds to the expected perspective.

### Statistical analysis

Statistical analyses were conducted using R version 4.5.0. The NDD and TD groups of participants were first described. Quantitative variables were summarized as mean and standard deviation, or alternatively as median with first and third quartiles depending on the distribution. Categorical variables were reported as counts and percentages.

The spontaneous perspective (egocentred vs. decentred) was first compared between the two groups (TD vs. NDD) using binomial logistic regression models adjusted for age and sex (male vs. female). Its dominance was analysed with linear regression models adjusted for the same covariates.

Then, in sessions two and three, differences in accuracy and response time between the two groups were tested using linear regression models adjusted for age, sex, and spontaneous perspective. Analyses were repeated within the TD group only, in order to assess the effects of age, sex and spontaneous perspective on accuracy. This additional exploratory analysis was not run for the NDD group, given the small sample size, sample heterogeneity, and the very low and unbalanced number of girls in thus group.

Linear regression coefficients were tested using t-tests, or alternatively by bootstrap resampling (10 000 replications) when model assumptions were not met. Possible interactions between explanatory variables were evaluated based on theoretical considerations. As the analyses were exploratory, no correction for multiple testing was applied.

## Results

### The spontaneous perspective (S1)

For 72 (97%) participants in the TD group and 30 (91%) in the NDD group, a spontaneous perspective could be determined. The frequency of spontaneous perspective assignment did not differ between the two groups (Fisher’s exact test, p = 0.17).

In the TD group, 49 (68%) participants adopted the trunk-centred perspective, 10 (14%) adopted the head-centred perspective, and 13 (18%) the decentred perspective. In the NDD group, 18 (60%) adopted the trunk-centred perspective, 8 (27%) adopted the head-centered, and 4 (13%) the decentred perspective. Considering the type of spontaneous perspective (egocentric versus decentred) adopted by participants, there were no significant differences between groups (OR = 1.72, p = 0.4), nor in terms of age or sex. The dominance of the spontaneous perspective was found to increase with age (β = 0.05; p < 0.001) and there was no significant difference between the NDD group (Mean of spontaneous perspective dominance = 0.49) and the TD group (mean of spontaneous perspective dominance = 0.35) [β = −0.11; p = 0.14].

### Recognition performance during imposed perspective-taking, in terms of accuracy and RT (S2) (see Table 3).

**Table 3:** Multivariable models results for accuracy and response time (RT) in session 2.

Variable	Accuracy model β [95% CI]	p	Response time model β [I95% CI]	p
Group (NDD vs. TD)	−0.22 [−0.36; − 0.07]	0.006	164 [−164, 491]	0.3
Age (years)	0.07 [0.04;0.09]	<0.001	11 [−50, 72]	0.7
Sex (Male vs. Female)	−0.08 [−0.18;0.01]	0.091	21 [−263, 305]	0.9
Spontaneous perspective (Head-centered vs. Decentred)	−0.20 [−0.40;0.02]	0.068	737 [282, 1,192]	0.002
Spontaneous perspective (Trunk-centered vs. Decentred)	0.04 [−0.10;0.17]	0.585	83 [−266, 432]	0.6

For this analysis, the data of 87 participants, 63 TD children, and 24 children with NDD were included (see Table 4). 11 children of the TD group and 9 of the NDD group were excluded, because either no spontaneous perspective was identified, or participants were misallocated in session 2.

**Table 4:** Accuracy and Response time in TD and NDD groups in session 2.

Characteristic	TD N = 63	NDD N = 24
Accuracy	0.91 (0.54, 0.96)^1^	0.70 (0.29, 0.94)^1^
Mean response time	3.114 (2.890, 3.585)	3.618 (3.077, 4.031)

^1^Median (Q1, Q3).

**Fig 2:**
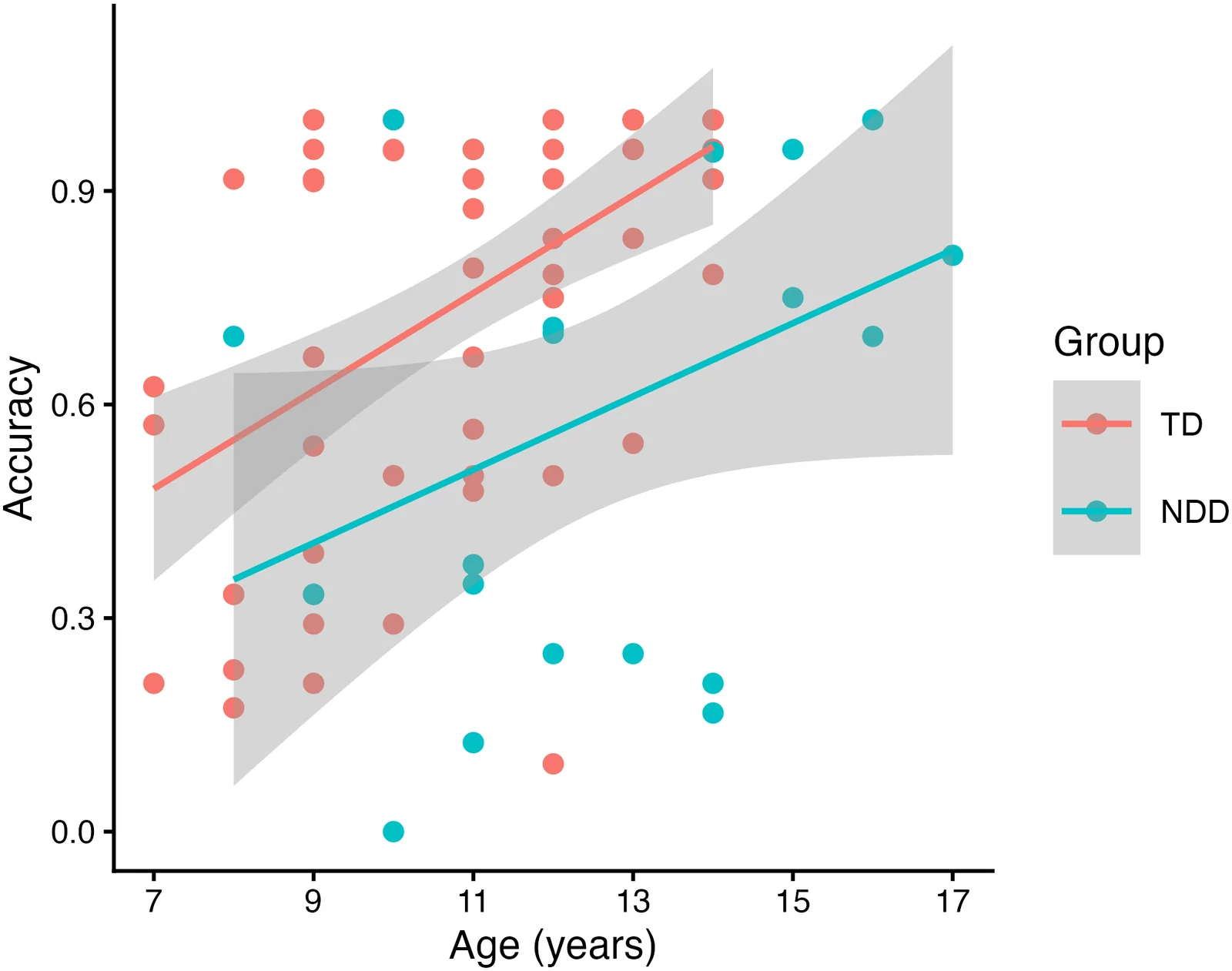
The evolution of accuracy according to age in the TD group and NDD group in Session 2.

Regarding accuracy (see Fig 2), participants’ accuracy was found to increase significantly with age for children in both groups (β = 0.07; p < 0.001). However, the children in the NDD group was significantly less accurate than that of the TD group (β = −0.22; p = 0.006). In addition, accuracy difference between boys and girls didn’t reach statistical significance (β = −0.08; p = 0.091). A lower accuracy was observed when the spontaneous perspective is head-centered compared to decentred, although the statistical evidence was weak (β = −0.20; p = 0.068). No significant statistical interaction was found between group and age (p = 0.3, Fig 2).

The models run within the TD group produced results consistent with those mentioned above, showing that accuracy significantly increased with age (β = 0.08; p < 0.001) and was significantly lower for boys than for girls (β = −0.12; p = 0.039). In addition, TD children with a head-centered spontaneous perspective exhibited significantly lower accuracy than TD children with a decentered spontaneous perspective (β = −0.26; p = 0.008). Similarly, TD children with a trunk-centered spontaneous perspective were significantly more accurate than TD children with a head-centered spontaneous perspective (β = 0.30; p < 0.001).

Regarding participants’ RT, NDD children didn’t significantly differ from TD children (p = 0.3). However, when the imposed perspective in S2 corresponded to a decentred spontaneous perspective, the RT of NDD children was significantly higher than that of TD children (β = 1131 ms, p = 0.004). In addition, children with a head-centered spontaneous perspective had a higher RT than those with a decentered spontaneous perspective (β = 737 ms, p = 0.002).

### Recognition performance when participants returned to their spontaneous perspective-taking (S3)

The statistical models included the data of 84 participants, 61 TD children and 23 children with NDD (see Table 5). Only the participants with a spontaneous perspective found in S1 and without a misallocation in S2 were included, hence 3 participants were removed. The results are summarized in Table 6.

**Table 5:** Accuracy and response time in TD and NDD groups in session 3.

Characteristic	TD N = 61	NDD N = 23
Accuracy	0.91 (0.78, 0.96)^1^	0.92 (0.29, 0.96)^1^
Mean response time	2,976 (2,700, 3,318)	2,967 (2,668, 3,481)

^1^Median (Q1, Q3).

**Table 6:** Multivariable models results for accuracy and response time (RT) in session 3.

Variable	Accuracy model β [95% CI]	p	Response time model β [I95% CI]	p
Group (NDD vs. TD)	−0.12 [−0.26;0.01]	0.075	−31 [−316;254]	0.8
Age (years)	0.06 [0.04;0.08]	0.000	−2.3 [−52, 56]	>0.9
Sex (Male vs. Female)	−0.07 [−0.16;0.02]	0.102	13 [−233, 258]	>0.9
Spontaneous perspective (Head-centered vs. Decentred)	0.12 [−0.09;0.34]	0.274	48 [−343, 439]	0.8
Spontaneous perspective (Trunk-centered vs. Decentred)	0.22 [0.07;0.37]	0.006	−471 [−772; −169]	0.003

Firstly, it should be noted that the perspective imposed in S3 corresponds to the spontaneous perspective computed in S1. The models showed a significant main effect of age (β = 0.06, p < 0.001). There was no significant difference between the two groups (β = − 0.12, p = 0.075). The numerical trend indicates a lower accuracy on average for the NDD group than the TD group. Accuracy was significantly higher when the spontaneous perspective was trunk-centered compared to decentred (β = 0.22; p = 0.006).

Regarding RTs, the age effect was higher on average by 157 ms/year for children whose spontaneous perspective was decentered, compared to those with a trunk-centered spontaneous perspective (p = 0.008). The age effect on RT was higher by 217 ms/year for children with a decentred spontaneous perspective compared to those with a head-centered spontaneous perspective (p = 0.008). Indeed, the models that RTs were significantly lower when the spontaneous perspective was trunk-centered than when it was decentered (β = −471 ms; p = 0.003). In addition, when the spontaneous perspective was decentered, RT was significantly higher for children in the NDD than in the TD group (β = 887 ms, p = 0.007). By contrast, when the spontaneous perspective was head-centered, RT was significantly lower in the NDD than in the TD group (β = −587.17 ms; p = 0.040).

## Discussion

Our study aimed to explore the developmental aspects of perspective-taking from a tactile modality in typically developing (TD) children and children with neurodevelopmental disorders (NDD), specifically ASD and DCD. To this end, we used the graphesthesia task developed in Auvray’s group [7,41,44]. In this task, participants are asked to interpret ambiguous tactile symbols traced by a matrix of tactile vibrators on their stomach. In session 1, participants were asked to adopt the perspective the most natural to them (spontaneous perspective), whereas in session 2 they were asked to switch to an imposed unspontaneous perspective, and in session 3 they were asked to switch back to their spontaneous perspective.

Firstly, consistent with the findings of Arnold et al. (2016) [44] in an adult population, we observed a similar distribution of spontaneous perspectives in both groups of children. For the TD group, 82% adopted an egocentric perspective (68% trunk-centred and 14% head-centred) while 18% adopted a decentred one. For the NDD group, 77% adopted an egocentric perspective (60% trunk-centred and 27% head-centred) and 13% adopted a decentred one. In line with our first hypothesis, children predominantly adopted an egocentric perspective. This result is also in line with Piaget’s theory (1967) [15] and several studies using the ambiguous symbol task (see [52] for a review). However, unlike Piaget (1967) [15], who emphasized that children are initially egocentric and somewhat unable to adopt an alternate perspective, in our experiment in the tactile modality, we found that children varied in the perspective they chose, with some of them preferring the head-centred, and others the trunk-centred or the decentred one. In addition, this variability differed as a function of the group (TD vs. NDD). Secondly, in both groups, children were able to switch perspectives in sessions 2 (see Fig 2) and 3. However, in line with Piaget’s hypothesis, there was an age effect concerning the dominance of the spontaneous perspective in both groups. This result is consistent with our second and third hypotheses.

Thirdly, and also in line with Arnold et al. (2016) [44], changing perspective in Session 2 incurs a cost, but returning to the spontaneous perspective in Session 3 induces a benefit. It should be noted that two different strategies can account for this cost. When an unnatural perspective was imposed on the participants, they may have identified the letter through an own-body transformation (i.e., a perspective taking process corresponding to the instruction). Alternatively, they may have maintained their spontaneous perspective and then mentally rotated the perceived letter (i.e., a mental rotation process, see Surtees et al. 2013 [10]). These two spatial processes can be either multisensory or visuo-spatial in nature. Additionally, there was a difference as a function of group. Indeed, during sessions 2 and 3, children with NDD had lower accuracy than those in the TD group. However, RTs were found to be quite similar, except for the imposed decentred perspective in session 2. In this case, the mean RT was significantly higher in the NDD group than in the TD group. In session 3, when the children returned to their spontaneous perspective, those with a spontaneous trunk-centred perspective had higher accuracy and faster RT than those with the two other perspectives. This result is in line with our final hypothesis.

Accuracy increased with age in sessions 2 and 3, which aligns with developmental improvement inhibitory and perspective-taking skills [6]. Similarly, lower performance in NDD is consistent with the numerous difficulties NDD patients exhibit in terms of perspective-taking and/or inhibition skills [54]. To assess the potential impact of including both ASD and DCD children in our NDD sample on the results, we conducted a sensitivity analysis excluding the 9 patients with DCD. The results showed similar profiles (see supplement materials 1).

There are other results worth discussing here. When the spontaneous perspective in session 1 was the head-centred one, the results revealed the following: i) Lower accuracy in session 2 for the TD group; ii) A trend towards a lower accuracy in session 3 than those who adopted a decentred perspective, for both groups; iii) In the NDD group, longer RTs in sessions 2 and 3 than those who adopted a decentred spontaneous perspective. The higher cost to switch from head-centred to decentred (and vice versa) than from trunk-centred to decentred (and vice versa) might be explained by the fact that the former requires both left-right and top-bottom inversions whereas the latter requires only the left-right inversion [55]. A substantial body of literature focusing on mental rotation abilities (i.e., involving tasks that depend on object-based spatial transformations) reveals a male advantage during childhood and adolescence (for a review see [56]). However, very few studies have focused on egocentric spatial transformation abilities. These studies did not find any gender differences [57–60]. The higher accuracy found for girls in our study could be explained by the larger number of boys in the NDD group.

Overall, our findings highlight the embodied strategy involved in perspective-taking, as well as its abnormalities in children with NDD. As previously mentioned, to interpret our results, it is necessary to consider several individual developmental dimensions, including cognitive, visuospatial, and global motor abilities [50]. In our task, the participants had to mentally simulate their own bodily movements to imagine themselves in another person’s position, in a process corresponding to a “rotation of the self” [61,62].

As was mentioned in the introduction, perspective-taking involves a conflict between two perspectives (our own and the other’s perspective). This conflicting information has to be overcome to successfully adopt another’s perspective. This begs the question of whether the perspective-taking ability is associated with executive functioning, more specifically with inhibitory control [16,63]. This latter is a key component of executive functions, which refers to high-level cognitive control processes that underlie flexible goal-directed responses to novel situations [64–66].

Abnormalities in inhibitory control may partly explain the lower performance of children in the NDD group. In a recent meta-analysis, Tonizzi et al. (2021) [67] found impairments in response to inhibition and interference control, differently affected by age and IQ, in children with ASD. Joyce et al. (2021) [68] also described these impairments, concerning motor and verbal responses, in children with DCD.

Heterogeneous results were found in studies focusing on Level 2 PT in ASD children. This could be partly explained by clinical heterogeneity among children with autism.

However, Pearson et al. (2013, 2016) [26,27] found that children with ASD struggled to perform egocentric transformations (which are the first step in completing Level 2 PT tasks) and relied on mental rotation strategies instead. Conson et al. (2015) [69] hypothesized that a decrease or lack of embodiment in ASD could be related to an inability to adopting someone else’s perspective. This could partly explain the lower performance of ASD children in our embodied task. According to Ogelman et al. (2013) [70], self-perception significantly predicts perspective-taking skills. Abnormalities in self-perception including impairments in agency, body ownership and interoception are described in ASD and linked to multisensory integration difficulties (for a review see [71]).

Orefice et al. (2024) [22] found that children with DCD had low scores in spatial perspective-taking than TD children aged 8–16 years old. Furthermore, the authors did not find any relation between age and improved in this population as confirmed in Gauthier et al.’s (2018) study [19]. Using a motor imitation task based on an interaction with a tightrope-walking avatar, the authors investigated whether participants in three groups (TD, ASD, DCD) can switch from an egocentric to a decentred perspective [19]. They found that the ASD group performed significantly better than the DCD group and that their performance improved with age.

### Limitations and perspective

To the best of our knowledge, this is the first study to focus on the developmental aspects of perspective-taking in the tactile modality in both typically developing children and those with a neurodevelopmental disorder (ASD and DCD). Due to high rates of comorbidity, frequent overlapping symptoms, and the limited number of children in our study, we decided to combine the ASD and DCD groups. Therefore, the study is exploratory. We conducted a sensitivity analysis to compare the performance of children in the TD and ASD groups (see supporting information files S1 and S2 Files). The results of this analysis were consistent with those presented in the result section, which related to the comparison between TD children and those with NDD. However, future research should compare perspective-taking performance of three groups of children (TD, ASD, and DCD) of a similar size, in order to highlight any specific abnormalities in the ASD and DCD groups. Given the influence of specific functions, such as inhibitory control, motor abilities, and visual-spatial abilities, on perspective-taking performance, the sample size should be large enough to assess the role of age, gender, and these developmental factors on performance in tactile perspective-taking tasks.

## Conclusions

Using a graphesthesia task, we explored the embodied strategy involved in perspective-taking in the tactile modality from a developmental perspective, and its specificities in children with NDD (ASD and DCD). TD children and children with NDD spontaneously adopted a plurality of perspectives, including a decentred one. However, they predominantly adopted an egocentric, trunk-centred, perspective. Their performance improved with age, and children in both groups were able to switch perspectives. However, children with NDD had lower performance than TD children. Several developmental impairments, including inhibitory control and multisensory integration amongst ASD, could explain our findings. Future research should compare perspective-taking performance of three groups of children (TD, ASD and DCD) of a similar and large enough size, to highlight any specificities in the ASD and DCD groups.

## Supporting information

S1 File Main characteristics of the participants. (DOCX)

S2 File Sensitivity analysis to compare the performance of children in the TD and ASD groups. (DOCX)
