# Supplementary material for: Spatial perspective-taking abilities on ambiguous tactile stimuli: The case of children with autism spectrum disorder or developmental coordination disorder
Source: PLoS One. 2026 Sep 21;21(9):e0355395. doi: 10.1371/journal.pone.0355395 (PMC13592701; doi:10.1371/journal.pone.0355395)
Supplement: Supplementary file 1 [file pone.0355395.s001.docx]

**Main characteristics of the participants**

**Table 1. Main characteristics of the participants.**

| Group | TD (N=74) | NDD (N=33) | |
| --- | --- | --- | --- |
| NDD type | Non relevant | ASD (N = 24) | DCD (N = 9) |
| Chronological age, mean (+/- SD) | 10,74 (1,99) | 12,04 (2,74) | 11,56 (2,70) |
| Male/Female | 32/42 | 20/4 | 8/1 |
| Right-handed/Left-handed | 65/9 | 17/3 | 9/0 |
| **WISC V**, mean (+/- SD) | Non relevant | 99,61 (18,16) | 94,14 (7,64) |
| Verbal Comprehension Index |  | 99,47 (23,34) | 105,00 (11,51) |
| Visual Spatial Index |  | 102,85 (14,77) | 94,00 (14,87) |
| Fluid Reasoning Index |  | 102,56 (23,43) | 100,875 (11,73) |
| Working Memory Index |  | 99,82 (18,30) | 88,75 (20,68) |
| Processing Speed Index |  | 89,65 (13,34) | 83,88 (16,42) |
| **ADI-R scores**, mean (+/-SD) [min – max] | Non relevant |  | Non relevant |
| Social impairment |  | 16,33 (4,24) [11 – 25] |  |
| Verbal communication |  | 12,28 (4,42) [5 – 20] |  |
| Restrictive, repetitive behaviors |  | 4,72 (2,05) [2 – 11] |  |
| **M-ABC2** mean (+/- SD) | Non relevant | Non relevant | 9,2 (5,54) |
| Comorbidity | Non relevant | ADHD (N = 8) | ADHD (N = 2) |

the robustness to the removal of the DCD children.
