# Supplementary material for: Spatial perspective-taking abilities on ambiguous tactile stimuli: The case of children with autism spectrum disorder or developmental coordination disorder
Source: PLoS One. 2026 Sep 21;21(9):e0355395. doi: 10.1371/journal.pone.0355395 (PMC13592701; doi:10.1371/journal.pone.0355395)
Supplement: Supplementary file 2 [file pone.0355395.s002.docx]

**Sensitivity analysis to compare the performance of children in the TD and ASD groups**

Table 1: Accuracy and Response time according to TD and TSA group in session 2

| **Characteristic** | **TD**   N = 63^1^ | **TSA** N = 19^1^ |
| --- | --- | --- |
| Accuracy | 0.91 (0.54, 0.96) | 0.70 (0.25, 0.96) |
| Mean response time | 3,114 (2,890, 3,585) | 3,579 (2,917, 4,044) |

^1^Median (Q1, Q3)

Table 1 represents the distribution of accuracy and mean response time (RT) observed in session 2 for the TD and TSA groups. No meaningful change was observed in the TSA group compared to the NDD group, showing that the results are robust to the removal of the DCD children.

Table 2: Multivariable models results for accuracy and response time (RT) in session 2

| **Variable** | **Accuracy model  β [95% CI]** | **p** | **Response time model β [I95% CI]** | **p** |
| --- | --- | --- | --- | --- |
| Group (TSA vs. TD) | -0.22 [-0.40;-0.04] | 0.018 | 52 [-299, 404] | 0.8 |
| Age (years) | 0.07 [0.04;0.09] | <0.001 | 11 [-53, 75] | 0.7 |
| Sex (Male vs. Female) | -0.08 [-0.18;0.01] | 0.090 | -12 [-300, 275] | >0.9 |
| Modal perspective (Head-centered vs. Decentred) | -0.21  [-0.41;0.01] | 0.061 | 788 [321, 1,255] | 0.001 |
| Modal perspective (Trunk-centered vs. Decentred) | 0.03 [-0.11;0.17] | 0.675 | 71 [-292, 434] | 0.7 |

Table 2 represents the results of the multivariable regression models for accuracy and mean response time (RT) in session 2. The adjusted group difference (TSA vs. TD) regarding accuracy is estimated at -0.22 (p = 0.018), close to the adjusted difference between NDD and TD (β = -0.22, p = 0.006). Similarly, the adjusted group difference of mean response time remains non significant in the sensitivy analysis. This shows that the analyses in session 2 are robust to the removal of the DCD children.

Table 3: Accuracy and Response time according to TD and TSA group in session 3

| **Characteristic** | **TD** N = 611 | **TSA** N = 181 |
| --- | --- | --- |
| Accuracy | 0.91 (0.78, 0.96) | 0.92 (0.71, 0.96) |
| Mean response time | 2,976 (2,700, 3,318) | 2,766 (2,519, 3,147) |

^1^Median (Q1, Q3)

Table 3 represents the distribution of accuracy and mean response time (RT) observed in session 3 for the TD and TSA groups. No meaningful change was observed in the TSA group compared to the NDD group, showing that the results are robust to the removal of the DCD children.

Table 4: Multivariable models results for accuracy and response time (RT) in session 3

| **Variable** | **Accuracy model  β [95% CI]** | **p** | **Response time model  β [I95% CI]** | **p** |
| --- | --- | --- | --- | --- |
| Group (TSA vs. TD) | -0.08 [-0.23;0.06] | 0.274 | -173 [-476;130] | 0.3 |
| Age (years) | 0.05 [0.03;0.08] | 0.000 | -1.8 [-58, 55] | >0.9 |
| Sex (Male vs. Female) | -0.06 [-0.15;0.03] | 0.197 | -29 [-274, 217] | 0.8 |
| Modal perspective (Head-centered vs. Decentred) | 0.12 [-0.10;0.35] | 0.295 | 117 [-280, 513] | 0.6 |
| Modal perspective (Trunk-centered vs. Decentred) | 0.24 [0.08;0.41] | 0.002 | -479 [-788; -171] | 0.003 |

Table 4 represents the results of the multivariable regression models for accuracy and mean response time (RT) in session 3. The adjusted group difference (TSA vs. TD) regarding accuracy is estimated at -0.08 (p = 0.274), while the one obtained for the comparison between NDD and TD is estimated at -0.12 (p = 0.075). This shows a slight decrease of the effect size and a lost of the trend towards significance. Conversely, the adjusted group differences of mean response time remain non significant in the sensitivy analysis, showing the robustness to the removal of the DCD children.
